# Advances in minimally invasive surgical techniques for lumbar disc herniation: a comprehensive review

**DOI:** 10.3389/fsurg.2025.1593195

**Published:** 2025-06-17

**Authors:** Weiqiang Lan, Hanbing Cui, Di Zhou, Xiao Xiao, Hongbo Xiong

**Affiliations:** ^1^ Department of Orthopedics, Chenggong District People’s Hospital, Kunming, Yunnan, China; ^2^ Operating Room, West China Xiamen Hospital of Sichuan University, Xiamen, China

**Keywords:** lumbar disc herniation, minimally invasive spine, spinal endoscopy, channel-assisted techniques, surgical treatment

## Abstract

Lumbar disc herniation (LDH) is a common and frequent disease in orthopaedics, which seriously affects patients’ physical and mental health as well as their daily life and work. There are various treatment methods for this condition, and different treatment plans should be adopted according to different situations. Traditional open surgery methods are relatively traumatic and have longer recovery times, while minimally invasive spine techniques have advantages such as smaller incisions, less bleeding, higher fusion rates, and faster recovery. This review summarizes the relevant literature on the application of minimally invasive techniques in the treatment of lumbar disc herniation in recent years, analyzes and compares the advantages and disadvantages of different approaches and endoscopic techniques, as well as reduction, decompression, and fusion effects. The aim is to provide reference for surgeons in selecting surgical procedures for the treatment of LDH.

## 1 Introduction

Lumbar disc herniation (LDH), a degenerative spinal disorder stemming from intervertebral disc pathology, is clinically characterized by localized low back pain accompanied by radicular symptoms in the lower extremities, typically resulting from mechanical compression and inflammatory irritation of spinal nerve roots (1). Current management of lumbar disc herniation (LDH) is principally categorized into conservative therapies and surgical procedures. Conservative therapies, including pharmacotherapy, physiotherapy, chiropractic, and traditional medicine can effectively alleviate clinical manifestations in most LDH patients (2). However, clinically significant symptoms may persist in some cases following conservative management (3). Notably, surgical intervention demonstrates superior clinical outcomes compared to non-operative approaches (4). Although conventional open surgery achieves effective decompression in lumbar disc herniation management, its limitations include significant invasiveness, higher intraoperative blood loss, and extensive disruption of spinal structures, leading to prolonged recovery times (5). In addition to the inherent trauma from surgery, these procedures involve extra risks related to complications from prolonged bed rest. While showing therapeutic efficacy, traditional open surgery has notable clinical drawbacks. Robot-assisted surgery is a relatively new technique in spinal surgery, and intraoperative image guidance significantly improves the accuracy of surgical procedures such as pedicle screw fixation and reduces pedicle screw displacement, providing benefits such as increased freedom during surgery, reduced incisions, elimination of hand tremors, and reduced surgeon fatigue, while reducing postoperative complications for patients (6, 7). With advancements in medical technology, minimally invasive techniques have rapidly evolved, offering favorable clinical outcomes, reduced tissue trauma, minimal blood loss, and accelerated postoperative recovery (8, 9). Recent developments in minimally invasive approaches have enabled effective removal of substantial nucleus pulposus tissue while maintaining optimal decompression efficacy, thereby addressing the limitations of conventional surgery. These technological innovations have increasingly established minimally invasive procedures as the preferred alternative to open surgery techniques (10).

There have been more literature reviews on the research progress of different access approaches for the treatment of lumbar disc herniation. However, the comparison of endoscopic techniques remains inadequate. Despite the growing adoption of minimally invasive techniques, further analysis is needed to compare endoscopic approaches and their long-term clinical outcomes. Therefore, this paper summarizes the literature related to the study of surgical treatment for lumbar disc herniation in recent years. It analyzes the advantages and disadvantages of access and endoscopic minimally invasive techniques in treating lumbar disc herniation and discusses and compares the decompression and fusion effects of the two techniques. The aim is to provide a reference for surgeons when choosing a treatment modality for lumbar disc herniation.

## 2 Methods

### 2.1 Search strategy

A systematic literature search was conducted on Web of Science and PubMed using MeSH/Emtree terminologies (e.g., lumbar disc herniation, minimally invasive spine, spinal endoscopy, X-LIF), along with synonyms and alternative spellings to ensure comprehensive coverage. Journal articles were limited to publication between 2005 and 2025, and in the English language. Additional articles were identified from reference lists of these publications. Articles were deemed relevant by title and abstract.

### 2.2 Inclusion and exclusion criteria

Original research articles were included that reported surgical management of LDH. Reports of cervical and thoracic discs were excluded, as were conference abstracts and reviews. Mechanistic LDH research was included. Data duplicated in different articles were identified and one article arbitrarily selected for inclusion. References to discectomy without mention of microscopic assistance or minimally invasive techniques were interpreted as open procedures.

## 3 Channel-assisted techniques

### 3.1 Posterior approach

The posterior lumbar interbody fusion (PLIF) procedure remains a cornerstone technique in spinal surgery, offering direct visualization of bilateral nerve roots and the dural sac (11). This approach enables comprehensive bilateral decompression, interbody grafting, and restoration of disc space height while enhancing stability of the anterior and middle spinal columns. Clinical studies confirm its high fusion success rates (>90%) and effective vertebral alignment correction (12, 13). A clinical series of 95 PLIF cases demonstrated solid fusion achievement in 84 patients (88.4%) (12), with robust fusion mass formation effectively preventing postoperative vertebral collapse (13). However, the PLIF technique carries inherent limitations: extensive dissection of paraspinal musculoligamentous structures elevates risks of neurovascular compromise and posterior column destabilization (14). Additionally, postoperative complications may include reduced lumbar lordosis and accelerated adjacent segment degeneration (15). Indications for PLIF include: discogenic low back pain, spinal stenosis, and failure of posterior posterolateral fusion, etc. Contraindications for PLIF include: severe osteoporosis with infection, severe epidural scarring, etc.

The transforaminal lumbar interbody fusion (TLIF), first proposed in 1981 as an improved technique over PLIF, allows direct visualization for interbody decompression and fusion, stabilizes the spine, minimizes structural damage to lumbar anatomy, and reduces intraoperative blood loss (16). In 2003, Foley et al. (17) introduced the minimally invasive TLIF (MI-TLIF), which has rapidly gained widespread clinical adoption. Utilizing the Kambin's triangle approach, MI-TLIF achieves adequate spinal canal decompression and interbody fusion (18). A meta-analysis by Heemskerk et al. (19) involving 16 studies (1,321 patients: 660 MI-TLIF vs. 661 open TLIF) with a minimum 2-year follow-up demonstrated that both techniques significantly improved pain relief, functional outcomes, and quality of life, with sustained effects at 2 years. Both approaches achieved high fusion rates (80.5% vs. 91.1%), indicating that MI-TLIF serves as an effective and safe alternative to open TLIF. MI-TLIF demonstrates superior clinical outcomes to open surgery, including sustained pain relief, higher fusion success, fewer reoperations/complications, and improved patient satisfaction. In a comparative study by Fang et al. (20) of 96 LDH patients with spinal stenosis (48 PLIF vs. 48 TLIF), both groups showed significant postoperative reductions in Numerical Rating Scale (NRS) scores for leg and back pain and improvements in Spitzer Quality of Life Index (SQLI) scores. However, TLIF demonstrated shorter operative times(106.24 vs. 138.73 min) and less intraoperative blood loss(375.24 vs. 608.28 ml) than PLIF. Indications for TLIF: Degenerative diseases of the lumbar spine with a positive discogram that is not associated with pathological changes in the spinal canal, reoperation of the lumbar spine or previous infections, and the formation of pseudoarthrosis between vertebrae. Particularly suitable for: single-segment lumbar disc herniation; with segmental instability or superolateral disc herniation. Contraindications to TLIF: narrowing of the intervertebral space by more than 5 mm compared to the normal intervertebral space, with degeneration of the discs of adjacent segments, with ossification of the posterior longitudinal ligament, with developmental stenosis of the spinal canal.

### 3.2 Lateral approach

The lateral lumbar interbody fusion (LLIF) technique involves the implantation of large intervertebral fusion cages to address various lumbar pathologies. This approach restores sagittal and coronal alignment, indirectly enlarges the neural foramina, and provides robust primary stability (21). A study by Nakashima et al. (22) involving 102 LLIF patients with ≥2-year follow-up demonstrated improvements in ligamentum flavum cross-sectional area and intervertebral disc dimensions, particularly at 6 months postoperatively. Systematic reviews by Lang et al. (23) of 1,080 cases revealed LLIF increased disc height by approximately 75%, while expanding neural foraminal and central canal areas by 36.4% and 25.4%, respectively. LLIF demonstrates substantial deformity correction capacity, achieving 10%-19% angular improvement (24), with reduced blood loss and complication rates compared to conventional deformity surgeries (25). This technique also prove effective in managing acute discitis, osteomyelitis, and lumbar trauma, with studies confirming complete infection eradication during follow-up in acute inflammatory cases (26). However, LLIF carries risks of lumbar plexus injury during psoas muscle dissection, particularly at L4-L5 levels. Its indirect decompression mechanism may result in inadequate neural element relief. Additionally, excessive endplate pressure during cage impaction and high interfacial friction during reduction maneuvers may lead to screw loosening, cage migration, and compromised fusion outcomes (27). Indications for LLIF: discogenic low back pain, mild-to-moderate lumbar spinal stenosis, degenerative scoliosis, lumbar instability, grade I-II lumbar spondylolisthesis, etc. Contraindications for LLIF: history of abdominal surgery with possible abdominal adhesions, severe lumbar spinal stenosis, vertebral body slippage at grade II or above, degenerative scoliosis combined with severe deformity, etc.

The oblique lumbar interbody fusion (OLIF) technique accesses the retroperitoneal space through the natural intermuscular planes between the left external oblique, internal oblique, and transversus abdominis muscles, utilizing the anatomical corridor between the aorta/inferior vena cava and psoas muscle to reach the intervertebral disc space. This approach allows complete disc space preparation for indirect decompression, interbody fusion, and internal fixation without transecting the psoas muscle, disrupting the lamina, paraspinal muscles, facet joints, or entering the spinal canal. OLIF effectively avoids lumbar plexus injury and demonstrates postoperative increases in foraminal height, lateral disc space height, and spinal canal area (28). In a retrospective study by Chen et al. (29) comparing 68 OLIF and 65 TLIF cases among 133 lumbar disc herniation patients, OLIF showed superior long-term pain relief, greater spinal alignment correction, earlier postoperative ambulation, shorter hospitalization, and faster recovery compared to TLIF. Similarly, Kotani et al. (30) compared clinical and radiological outcomes between OLIF and minimally invasive TLIF for lumbar degenerative diseases, with mean follow-ups of 51 and 69 months, respectively. The OLIF group exhibited better pain relief, lumbar function, gait recovery, and higher fusion rates (98% vs. 90%), suggesting OLIF as a preferable option for lumbar fusion candidates. However, OLIF carries risks of peritoneal tears, abdominal vascular injuries, and lumbar plexus damage (31, 32). Zhu et al. (33) analyzed 71 OLIF and 66 minimally invasive TLIF cases, reporting higher complication rates with OLIF(29.4% vs. 9.7%). Additionally, incomplete endplate preparation during OLIF may lead to graft nonunion or disc space collapse (34). Indications for OLIF: discogenic low back pain, lumbar spondylolisthesis (degree I or II), lumbar degenerative disease combined with moderate narrowing of the central lumbar canal or foramina, degenerative lumbar scoliosis, and adjacent segment degeneration after lumbar spinal fusion. Contraindications to OLIF: Severe central spinal stenosis and a high degree of spondylolisthesis, severe osteoporosis, long-segment scoliosis, and a history of abdominal surgery.

### 3.3 Anterior approach

The traditional anterior lumbar interbody fusion (ALIF) technique involves complex surgical maneuvers with inherent risks of vascular and neural injuries. In contrast, minimally invasive ALIF employs small abdominal incisions or laparoscopic approaches to access the lumbar spine, enabling extensive discectomy and large cage implantation while maximizing endplate coverage and preserving the posterior ligamentous complex. Compared to posterior approaches, minimally invasive ALIF achieves effective anterior spinal column reconstruction through convex-side cage placement, better restoring lumbar lordosis and intervertebral height with superior fusion rates (35). A retrospective study by Kuang et al. (36) comparing 42 mini-open ALIF and 40 TLIF cases in lumbar disc herniation patients demonstrated shorter operative times and reduced blood loss in the ALIF group. Rathbone et al.'s meta-analysis (37) revealed ALIF's advantages over posterior approaches (PLIF/TLIF) in operative duration, intraoperative hemorrhage, and clinical outcomes (VAS and ODI scores). Glassman et al.'s multicenter study (38) further confirmed ALIF's superior SF-36 and ODI outcomes at 1- and 2-year follow-ups compared to TLIF/PLIF. ALIF complications primarily include vascular injuries and retrograde ejaculation. Bateman et al.'s systematic review (39) of mini-open retroperitoneal ALIF reported arterial and venous injury rates of 0.06% and 1.65%, respectively. Retrograde ejaculation occurs in 2% of retroperitoneal vs. 25% of transperitoneal laparoscopic approaches (40), with mini-open retroperitoneal ALIF showing a 0.87% incidence (39). ALIF demonstrates optimal safety at L5-S1 levels, with escalating vascular risks at proximal segments. Consequently, ALIF is generally reserved for L4-S1 disc pathology, while its application at higher lumbar levels remains controversial. Indications for ALIF: middle and lower lumbar disc herniation, lumbar spondylolisthesis up to grade II, anterior stabilisation. Contraindications for ALIF: posterior spinal column structural variations, lumbar spondylolisthesis above grade II, infections, severe osteoporosis, abdominal adhesions.

## 4 Endoscopic techniques

Recent advancements in endoscopic systems and minimally invasive surgical instrumentation have significantly propelled the development of endoscopic lumbar decompression and fusion procedures. Broadly, spinal endoscopic systems are categorized into gas-medium and water-medium systems. Gas-medium systems employ fixed microendoscopes attached to tubular cannulas for visualization, eliminating the need for continuous saline irrigation (41). Technological evolution has facilitated the clinical application of hybrid water-gas microendoscopic systems. Water-medium systems are further classified into single-channel and dual-channel endoscopic systems. Single-channel spinal endoscopic techniques utilize a coaxial working cannula integrating both visualization and operative instruments, yet face limitations in visualization range and operational efficiency. In contrast, dual-channel spinal endoscopic systems separate the endoscope and working instruments into independent channels, providing enhanced visual clarity and expanded operational mobility.

### 4.1 Microendoscopic discectomy (MED)

MED shares analogous surgical principles and procedural steps with conventional open discectomy but demonstrates reduced invasiveness. This technique achieves spinal canal decompression through nucleus pulposus removal under microendoscopic visualization (42). Notably, MED specifically refers to the surgical procedure rather than the endoscopic system itself, and should not be conflated with microendoscopic technology (4). A long-term follow-up study by Masuda et al. involving 1,968 LDH patients (646 MED vs. 1,322 minimally invasive/open discectomy cases) revealed comparable short-term reoperation risks within 90 days postoperatively. However, MED exhibited a higher 5-year cumulative reoperation rate (12% vs. 7%), suggesting greater recurrence potential compared to minimally invasive or open discectomy approaches. Indications for MED: single-level LDH. Contraindications for MED: concomitant lumbar instability, spinal canal stenosis, or previous lumbar surgeries (43).

### 4.2 Single-channel spinal endoscopic

Single-channel spinal endoscopic procedures require only 7–10 mm incisions and minimize muscle/soft tissue dissection, thereby reducing surgical trauma (44). This technique can be categorized into coaxial large-channel or small-channel endoscopic systems. The large-channel system provides a wider operative field for efficient surgical maneuvers, while the small-channel system's restricted instrument dimensions may lead to inadequate disc space preparation and prolonged operative durations.

Percutaneous endoscopic lumbar discectomy(PELD) and percutaneous endoscopic interlaminar discectomy (PEID) are two principal modalities of Interlaminar endoscopic lumbar discectomy (IELD) (45). PELD, a less invasive alternative to MED, utilizes transformational endoscopic technology and represents a revolutionary advancement in minimally invasive spine surgery. Zheng et al. (46) demonstrated that single-channel PELD offers advantages over open surgery in lumbar disc herniation (LDH) management, including shorter operative time, reduced blood loss, minimized incision size, accelerated postoperative mobilization, and comparable complication rates. PELD is indicated for foraminal, central, and recurrent LDH subtypes (47).

PEID employs a posterior approach through the interlaminar space, mirroring conventional open posterior techniques (48). Xu et al. (49) reported PEID's superior efficacy, simplified needle positioning, shorter operative duration, and minimal impact on lumbar stability compared to PELD, particularly advantageous for patients with high iliac crests (wide sacroiliac spacing) or L5 transverse process hypertrophy where transforaminal access is challenging. However, PELD carries elevated risks of nerve root irritation and intraoperative discomfort. Indications for PEID: axillary and sequestrated LDH (50).

Numerous studies have demonstrated comparable clinical outcomes between various single-channel percutaneous endoscopic lumbar interbody fusion (PELIF) techniques and conventional channel-assisted approaches in treating lumbar disc herniation (LDH), with no significant differences in mid- to long-term efficacy. Regarding clinical effectiveness, a retrospective analysis by Butle et al. (51) involving 100 patients revealed significant reductions in Oswestry Disability Index (ODI) following coaxial endoscopic fusion. Similarly, Nakamura et al. (52) documented marked improvements in Visual Analog Scale (VAS) scores for low back pain, leg pain, and ODI postoperatively. These findings collectively indicate satisfactory clinical efficacy of coaxial endoscopic fusion. However, single-channel spinal endoscopic techniques exhibit inherent limitations (51), including restricted indications, prolonged operative and fusion times, risks of cage migration, nerve root injury, and a steep learning curve. Jacquot et al. (53) reported postoperative complications in 57 PELIF cases: cage migration occurred in 13 patients (22.8%), while nerve root injury manifested as aggravated pain or sensory abnormalities in 8 cases (14%). Although single-channel endoscopic techniques offer certain advantages, their limitations necessitate careful consideration. Therefore, surgical approach selection should involve comprehensive evaluation of individual patient characteristics and disease progression.

### 4.3 Biportal spinal endoscopy

First reported in 2016 as a dual-channel decompression technique (54), the unilateral biportal endoscopic (UBE) approach was later formally established (55). UBE utilizes independent optical and working channels with continuous saline irrigation, providing enhanced visualization and instrument maneuverability in confined surgical spaces. This system is classified into unilateral and bilateral biportal endoscopic approaches. Compared to traditional posterior surgeries, UBE-assisted lumbar interbody fusion facilitates faster recovery in patients with degenerative lumbar conditions (56).A prospective case-control study by Liu et al. (57) comparing unilateral biportal endoscopic lumbar interbody fusion (ULIF) with PLIF demonstrated reduced intraoperative blood loss, postoperative drainage volume, and shorter hospitalization in the ULIF group. ULIF also showed superior leg pain VAS scores and JOA scores at postoperative day 5 compared to PLIF. Complications of UBE include dural tears, nerve root injury, adjacent segment degeneration, cage migration, and chronic low back pain due to paraspinal muscle atrophy or fibrosis (58). The incidence of dural/nerve injuries is slightly higher than in minimally invasive TLIF, while cage migration rates exceed those of minimally invasive TLIF (37). Kim et al. (59) observed a 1.6% dural tear rate in 1,551 UBE cases, suggesting cage migration may relate to intraoperative endplate damage and irrigation. Although UBE is minimally invasive, it causes greater tissue disruption than PELD/PEID. However, its broad visualization and operational flexibility make it suitable for complex spinal pathologies, complementing transformational endoscopic techniques.

In decompression, both single- and biportal endoscopy allow full visualization. However, single-channel endoscopic fusion requires partial blind manipulation during endplate preparation and cage placement under nerve retractor protection, often necessitating fluoroscopy (37). In contrast, UBE permits complete endoscopic visualization during endplate resection and cage implantation, improving precision and fusion rates. While UBE reduces tissue trauma compared to open surgery, it is more invasive than single-channel endoscopy. The UBE transarticular or interlaminar approach requires complete facet joint resection to expose Kambin's triangle, which is describe as that the triangular working zone is bordered anteriorly by the exiting root, inferiorly by the proximal plate of the lower lumbar segment, posteriorly by the proximal articular process of the inferior vertebra, and medially by the traversing nerve root and dural sac, whereas single-channel endoscopy achieves sufficient exposure without full facetectomy, preserving protective articular and ligamentous structures (24, 60).

## 5 Conclusion

Minimally invasive spine has advanced significantly and is rapidly evolving. Its treatment methods are diverse, each with its own advantages and disadvantages. The anterior approach was first proposed, but due to its abdominal approach, it can cause more complications and greater surgical risks. The posterior approach and the transforamen approach are classical lumbar fusion surgery methods and are widely used in clinical practice. The lateral approach can achieve good clinical efficacy and has unique advantages in spinal orthopedics, discitis, osteomyelitis and lumbar trauma. However, due to the small minimally invasive passages and limited access and operating space for surgical instruments, it may lead to limited vision of the surgeon during observation and operation, which limits the feasibility of some complex slip-off orthopedic surgery requiring larger surgical instruments. In addition, minimally invasive channel technology also has insufficient end plate preparation, easy to cause muscle and nerve damage under non-visual operation, long intraoperative fluorescence time, and large radiation dose. Performing critical surgical operations under visualization with endoscopy is safer, effectively reducing the incidence of related complications and early postoperative pain. However, minimally invasive endoscopy still faces challenges such as a steep learning curve, high surgical difficulty, limited surgical indications, prolonged surgery times, and high equipment costs (61). Through comparative research and summary of various surgical methods, we can better understand their development history, understand their principles of action, clarify their clinical efficacy, etc., and provide certain reference for the development trend of future treatment methods.

As a typical representative of the “new technological revolution” in the medical field, orthopedic surgical robot is the core intelligent equipment to promote the development and popularization of precision and minimally invasive surgery. Robotic surgical devices can work seamlessly with navigation systems and are well integrated into minimally invasive spine surgery techniques. In addition to simplifying the placement of pedicle screws and other spinal implants, spinal robots can also assist surgeons in planning the trajectory of tubular retractor, stabilize the retractor with a mechanical arm, and plan the placement of interbody fusion cage (62). With the continuous progress of spinal robot technology, more capabilities such as bone decompression can be mastered by robots in the future, so as to help surgeons to complete surgery to a greater extent.

Despite their advantages, minimally invasive approaches face challenges such as a steep learning curve for surgeons, extended operative times during the initial learning phase, and high costs of specialized equipment, which may limit accessibility in resource-constrained settings.

In summary, minimally invasive surgery offers numerous advantages in treating lumbar disc herniation and can provide patients with safer and quicker treatment options. With advancements in robotics and navigation technology, lumbar surgery is poised to become more precise, individualized, and accessible, ultimately flattening the steep learning curve associated with minimally invasive techniques. With the popularization of day surgery, the combination of basic minimally invasive techniques can meet the needs of the vast majority of lumbar disc herniation, accelerate the rehabilitation process of patients, and ultimately reduce the burden and cost of patients and medical institutions.
